# Physical Chemistry Models for Chemical Research in the XXth and XXIst Centuries

**DOI:** 10.1021/acsphyschemau.3c00057

**Published:** 2024-02-09

**Authors:** Josep M. Ribó, David Hochberg

**Affiliations:** † Department of Inorganic and Organic Chemistry, University of Barcelona, c. Martí i Franquès 1, 08028 Barcelona, Catalonia, Spain; ‡ Institute of Cosmos Science (IEEC-UB), c. Martí i Franquès 1, 08028 Barcelona, Catalonia, Spain; § Department of Molecular Evolution, Centro de Astrobiología (CSIC-INTA), E-28850 Torrejón de Ardóz, Madrid, Spain

**Keywords:** chemical complexity, chemical syllabus, general evolution criterion, nonlinear irreversible thermodynamics, stoichiometric network analysis

## Abstract

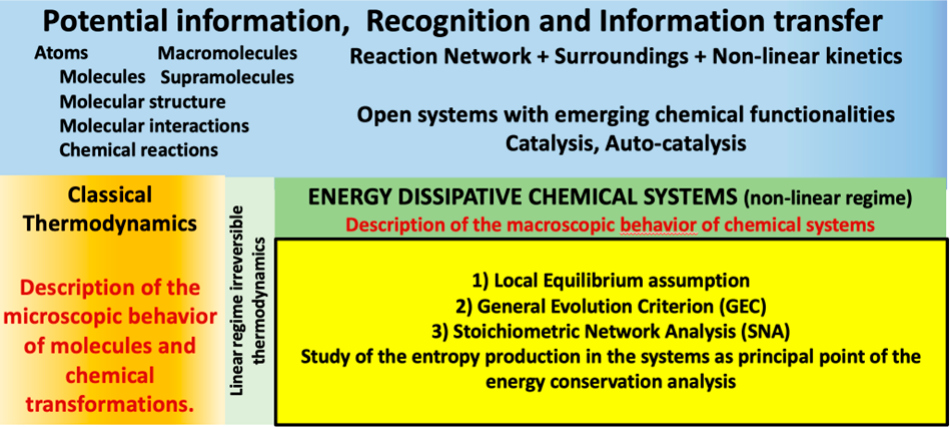

Thermodynamic hypotheses and models are the touchstone for chemical results, but the actual models based on time-invariance, which have performed efficiently in the development of chemistry, are nowadays invalid for the interpretation of the behavior of complex systems exhibiting nonlinear kinetics and with matter and energy exchange flows with the surroundings. Such fields of research will necessarily foment and drive the use of thermodynamic models based on the description of irreversibility at the macroscopic level, instead of the current models which are strongly anchored in microreversibility.

## 1 Introduction

The number of reports in physical chemistry journals dedicated to nanochemistry, self-assembly, and supramolecular chemistry is steadily increasing. Editorial objectives to promote such type of reports are well-intentioned because these reports deal with rarely studied aspects of chemistry. Many of these reports present dramatic results whose novelty is precisely in their disagreement with the current thermodynamic models based on classical reversible thermodynamics. In spite of this, they lack the kinetic and energetic analyses required for their thermodynamic justification. Furthermore, the thermodynamic models contradicted by these results are generally maintained as being valid in the discussion of the results. Theories, in a metaphysical sense, have been considered hypotheses or models, whose completeness can only be proved through their falsification,^1^ i.e., in their disagreement with experimental facts. It is through their confrontation with experimental results that disagreement is detected. Objectivity in chemical research is required not only to search for clear contradictions with the dominating theories but also to use the confirming results for the development of more specific topics and for applied development and engineering, through skillfully designed experimental results. Notice that to make effective progress, both in applied and theoretical chemistry, the basis of robust hypothesis/model building is a necessary condition. Experimental results, when they are not erroneous or simply fraudulent (see, for example, ref (2)), are true verifiable empirical facts independent of their agreement or contradiction with the current models. Historically, because of the confusion of *Principles* and *Fundamental Physical Laws* with methodological hypothesis, chemists have had a tendency to skepticism in believing experimental results that contradict the current accepted models. A paradigm of this is the case of Staudinger’s foundation of macromolecular chemistry (see ref (3)).

This Perspective expresses the authors’ opinion about how the extended, and fruitfully used, thermodynamic models based on microreversible thermodynamics are proving to be inadequate for the study of the experimental reports dealing with complex open systems. In consequence, a qualitative conceptual change is expected to occur in the physical chemistry publications in the second half of the 21st century. By “chemical complexity” we understand the interaction of a nonlinear reaction network with its surroundings, through independent and coupled transformations and chemical flows showing nonlinear dynamics, emergence of new behaviors, and functionalities, such as is defined in refs (4−6).

Chemistry experiments, except for some specific methodologies such as analytical titrations, belong to the domain of nonlinear irreversible thermodynamics. Furthermore, most chemical experiments obtained by changing the reaction parameters and boundary conditions yield, under the so-called thermodynamic control, a final nonequilibrium stationary state (NESS) that forms part of a continuous extension of the equilibrium state (see pp 410–411 in ref (7)) or to kinetically trapped states, in the case of the so-called kinetically controlled experiments. Both scenarios can be interpreted considering the relationships between equilibrium constants and reaction rate constants. The stable NESS’s that belong to the continuous extension of the thermodynamic equilibrium make up what is called the thermodynamic branch of the system. In the case of kinetic and dynamic nonlinearities, and of strong coupling of the reaction network with the matter and energy exchange with the surroundings, i.e. for complex chemical systems, the NESS of the thermodynamic branch may become unstable, and this conceptual continuum extension between irreversible and equilibrium thermodynamics is broken. In this new scenario,^8^ the system may evolve to a new stable NESS, but also to oscillatory states, and even to full chaotic behavior. This theoretical description belongs to the topic of energy dissipative systems. A simple example of this is the case of spontaneous mirror symmetry breaking (SMSB) where, instead of the expected racemate of enantiomers in a 1:1 ratio, the final state is that of a practically 100% enantiomeric excess.^9^

The theory of irreversible thermodynamics, able to be applied to the majority of open chemical systems, has been established for a long time.^10,11^ Furthermore, some authors have recognized the importance of nonlinear chemical dynamics and of reaction-diffusion phenomena in modern chemistry.^12,13^

With respect to the chemistry underlying the topic of the origin of life, Eschenmoser and Kisakürek have written: “Regarding the thermodynamic and kinetic prerequisites, eminent physical chemists active in the field of self-organization theory of organic matter (M. Eigen, I. Prigogine, H. Kuhn, and others) have paved the way for the organic chemists, not in the least psychologically.”^14^ Notice that autocatalysis and cooperative phenomena are characteristic of the new type of reports published in physical chemistry journals. Models based on irreversible thermodynamics are necessary for the interpretation, design, and dissemination of chemical complexity. It will be necessary to change the present pedagogical paradigms and academic syllabus in thermodynamics and chemical kinetics and, last but not least, to surmount ingrained psychological barriers.

## 2 Thermodynamic Models That Supported XX Century Chemistry

Chemical progress during the 20th century has been supported by hypotheses expressed in two physicochemical “models”: (a) and (b). These are as follows.

### 2.1 (a) Quantum Chemical Geometrical Description of Molecular Structure

Through the approximations of quantum mechanics, molecules can be described as geometric structures. This also yields a description of localized molecular bonds, their energies and electronic distributions, and fundamental physical properties of molecules. Pedagogical qualitative representations, especially those presented by L. Pauling,^15^ relating molecular bonds and electron distributions with respect to a positively charged nucleus, have served chemists to establish a basic description of the properties of organic functional groups, thus converting Chemistry from a catalog of properties and experimental procedures to a logical and pedagogically scientific field. In this respect, there is a dramatic difference between the most successful organic chemistry textbooks of the first and second halves of the XX century. This may be appreciated for example, by comparing the books of Karrer^16^ and of Cram and Hammond,^17^ whose last and first editions, respectively, were both published in the same year 1959. Furthermore, the structural quantum chemical model, through the bond structure, also explains stereochemical isomerism. From the point of view of a historical evolution of chemical knowledge, it is significant that K. Mislow, who established a clear chemical description of molecular chirality,^18^ which is nowadays used in all chemistry syllabuses, worked on his Ph.D thesis under Pauling’s supervision.

### 2.2 (b) Relationship between Energetics and Reaction Rates Derived from the Transition State Theory

The experimental fact that chemical reactions possess an energy barrier, described by the Arrhenius equation, was theoretically related to the Gibbs free energy by the Eyring formulation.^19^ From this and other seminal contributions (e.g., the so-called Bell–Evans–Polanyi principle),^20^ the reaction path model of the chemical transformation (reaction coordinate model) was derived. This allowed one to relate kinetic experimental data for specific reactions in “homologous” families of organic compounds and to build, using the structural models of (a),^21,22^ the types of reactions and their mechanisms. The reaction mechanism methodology has been so successful that a general scheme of reaction mechanisms has been built that can be applied to nearly any organic chemical reaction, without further experimental confirmation. For an example of today’s practically forgotten methodology to relate kinetic data with reasonable molecular reaction mechanisms, see ref (23).

The interplay between the above quantum chemistry and reaction coordinate models is expanded today to the calculation of complex potential energy surfaces of high molecular weight species and supramolecular systems and substrates systems. However, the most impressive example of the successful convergence of models (a) and (b) was in the explanation of the breaking of the Bell–Evans–Polanyi hypothesis,^20^ relating reaction energy with reaction rates, and for some specific reactions (pericyclic reactions).^24^ This paved the way to reveal the significance of topology and symmetry of molecular orbitals, of the activated complex at the transition state.^25,26^ This milestone correlated not only molecular structure but also the distribution and energy of the more accessible levels of the electrons in molecules for explaining chemical reactivity. It is also worth pointing out, that this is a case study of how scientific progress may occur through several diverse contributions, but that the glory of discovery goes only to a chosen few.^27^

No less important are the results confirming hypotheses (a) and (b) that have led to a high degree of predictability of the chemical organic reactions allowing the retro-synthetic analysis in chemical synthesis. However, this predictability assumes that the output of the synthesis shows a composition that is an extension of that expected from chemical equilibrium. However, this cannot be extrapolated to complex open thermodynamic systems, which have nonlinear kinetics and strong coupling with matter and energy flows with the surroundings.

Notice that all the former models or chemical paradigms belong to a description rooted in time-reversible classical mechanics or else in Schrödinger’s quantum physics and do not consider the irreversible evolution predicted by thermodynamics and its second principle.^11^

## 3 Necessary Elements for New Models to Aid Research in Chemical Complexity

Theoretical research in thermodynamics is today centered in “extended thermodynamics”, which considers the local equilibrium assumption, or approximation, not to be valid. This is the case of some common processes as polymer relaxation and gel dilation and of the physical and cosmological scenarios of ultrasonic propagation, shock waves, nuclear collisions, and gravitational collapse (blackhole formation). However, local equilibrium is a common chemical scenario in irreversible thermodynamics (see pp 14–16 of ref (10)) and can be applied to most chemical processes, with the exception of a few phenomena (see section 4.2).

The reason why few theoretical reports are being produced in irreversible thermodynamics under the local equilibrium assumption is a consequence of the completeness of the theory developed by the Brussels school in the middle of the XX century, where the seminal and pedagogical report on this is the book of ref (10). However, the paradox is that this theoretical work, despite being universally accepted, is rarely used to justify thermodynamically experimental results obtained in far from equilibrium irreversible scenarios.

**Scheme 1:**
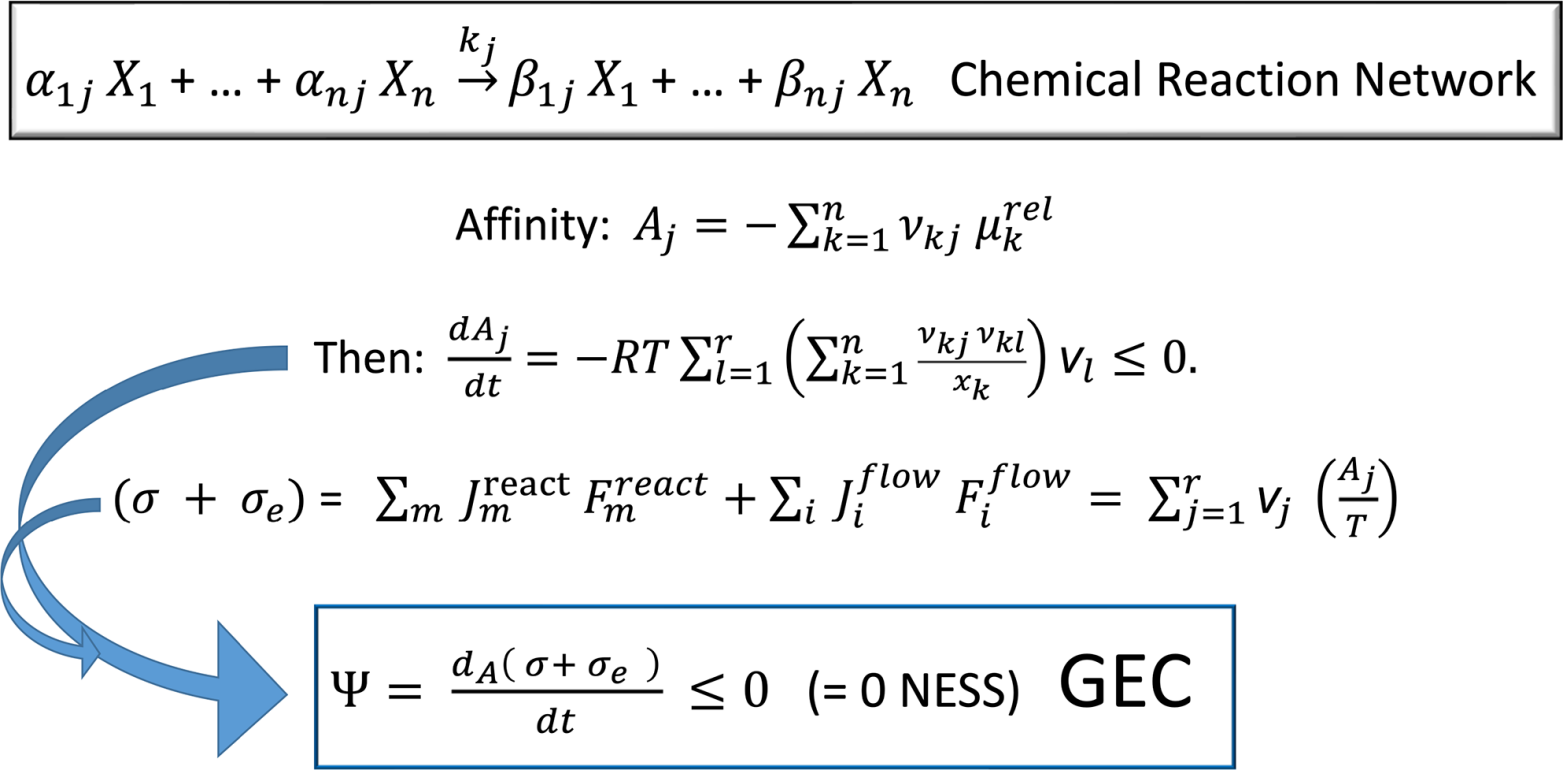
Basic Steps Leading to the Demonstration of the General Evolution Criterion (GEC) Extended to Include Input and Output Flow of Chemical Species to an Open Flow System Such As That of Figure 1 Start from a general network of *j* = 1,2,···,*r* one-way chemical reactions involving *n* species *X*_*j*_ in which the forward and reverse transformations are treated individually encompassing matter fluxes into, and out from, the reactor. The stoichiometric coefficients define the stoichiometric matrix: ν_*kj*_ = (β_*kj*_ – α_*kj*_) (see Section 4.4.2). The time derivative of the affinity *A*_*j*_ yields a manifestly negative semidefinite expression, where *x*_*k*_ is the concentration of the *k*th species and *v*_*l*_ is the reaction rate for the lth reaction. The sum of the entropy production plus the entropy flux (σ + σ_*e*_) can be written in the canonical form as products of thermodynamic forces *F* times flows *J*, eq 1, and then more concretely in terms of one-way reaction rates times affinities, as shown above. Then the extended GEC immediately follows. The GEC is the statement that the derivative of the entropy production plus entropy flux with respect to temporal changes in the affinities *A* (the chemical forces) is always negative and is equal to zero only at a NESS. It reduces to the standard GEC^10^ of Glansdorff and Prigogine for chemical systems lacking entropy fluxes: σ_*e*_ = 0.

The decision to be taken on which thermodynamic scenario is applicable to a specific experimental result can be arrived at by answering the following questions in hierarchical order (see also Scheme 1): (i) Do the results belong to thermodynamic equilibrium or to an irreversible scenario? (ii) When the experimental results correspond to an irreversible scenario, the question is if they belong to the linear regime or to the nonlinear regime. The latter occurs for affinity/*RT* ≫ 1.^28^ (iii) If the results belong to the linear regime of irreversible thermodynamics, the final NESS can be considered as a continuous extension of equilibrium (the thermodynamic branch), and the current models of the reaction paths (Section 2) can be applied. (iv) If the system belongs to the nonlinear regime, it is necessary to distinguish whether the approximation of local equilibrium is valid or not. Section 4.2 analyzes the meaning of local equilibrium giving a general criterion to decide on this. In the case that the local equilibrium approximation is not valid, the system belongs to the domain of extended thermodynamics.^29^ We think that the latter case, and in the approaching horizon of 2050, will remain within the theoretical range of physical chemistry research. (v) When in (iv) the local equilibrium approximation is applicable, then whether, or not, the final nonequilibrium stationary state (NESS) corresponds to the thermodynamic branch of possible stationary states should be analyzed (see section 4.3). In the first case, the NESS’s are a continuous extension of the thermodynamic equilibrium by virtue of the boundary conditions and the results, at least qualitatively, would be not in flagrant contradiction with thermodynamic models based on microreversibility. (vi) NESS’s which do not belong to the thermodynamic branch occur beyond a critical value of the entropy production which destabilizes the NESS of the thermodynamic branch. The negative answer in (v) implies a bifurcation scenario where NESS’s, other than those of the thermodynamic branch, emerge. The emerging NESS’s can be detected by graphical representations of the final states obtained by changing reaction and system parameters (see, e.g., ref (30)). This is greatly facilitated by the use of the matrix representation of the reaction flows [stoichiometric network analysis (SNA); section 4.4.2). The stability of the NESS is evaluated by the analysis of the corresponding Jacobian, but that is possible analytically only in systems with few variables, i.e., with few chemical species. For systems with many variables and parameters, the Jacobian stability analysis can always be carried out numerically. The final NESS’s composition and their stability cannot be predicted from the chemical potentials of the species of the system, despite the fact that they determine the thermodynamic constraints, expressed in the microreversible models between the equilibrium constants and reaction rate constants.^31^ However, the stationary states must fulfill the balance between the exchange entropy and the internal entropy production (section 4.1). Furthermore, the evolution dynamics of the system, under the local equilibrium assumption, is determined by General Evolution Criterion (GEC; section 4.1), which represents the macroscopic behavior arising from the irreversibility. GEC applies both to reversible as well as irreversible thermodynamics, but the linear relationship between the generalized thermodynamic forces and currents, that is valid only in the linear regime of irreversible thermodynamics, had most likely obscured its fundamental role in the evolution of systems in the far from equilibrium nonlinear regime.

Notice that since the entropy production is the sum of products of the forces (affinity in a chemical reaction) times their currents, it increases with the species concentrations. Therefore, for a chemical system having linear kinetic dependences, the critical entropy production leading to the instability of the thermodynamic branch would first occur at very high concentrations, this means at large densities and viscosities. This commonly leads to reaction-diffusion controlled kinetics, so that either spatial dissipative structures or full chaotic outcomes occur. However, systems showing high nonlinear kinetic dependences as complex autocatalytic reaction networks, and polymerizations or aggregations to nanoparticles^32^ showing cooperative effects, may lead to bifurcations. It can be argued that nonlinear behaviors are not common in chemistry, but this is only true when one considers the well-developed present chemistry in solution, but not chemistry in complex systems where cooperative and autocatalysis easily arise.

### 3.1 Dissipative Spatial-Temporal Structures Arising from Competition between Reaction and Diffusion

Nowadays significant reports in physical chemistry journals correspond to reports on spatial dissipative structures. They are justified by reaction-diffusion scenarios^13,33−35^ which are free from controversies. However, the results based on the emergence of bifurcations in homogeneous solutions, such as the racemic biases emerging in spontaneous mirror symmetry breaking processes (SMSB),^9,36^ or the self-assembly of nanoparticles, where it is not possible to follow visually the different nanoparticle morphologies, still lead to skepticism in most chemists regarding justifications based on irreversible thermodynamic bifurcation scenarios. An historical example of this is the Belousov–Zhabotinsky (BZ) reaction, well-known to all chemists for yielding dramatic chiral spatial figures, but in fact, the rejection of the initial Belousov report by peer reviewers was made on the argument that oscillatory behavior should not be expected in homogeneous solutions, which was the media used in the original report.^37^ Nevertheless, a number of authors are now considering the bifurcations arising in an irreversible thermodynamic scenario for justifying the emergence of NESS’s, of oscillatory phenomena, and the self-assembly of supramolecular systems.^38−42^

Results on spatial-temporal dissipative structures are mostly justified by considering that the increase of the absolute rates to achieve the critical point of the emergence of dissipative structures originates from effects acting upon the species migration. This being true, it overlooks the fundamental cause because the currents are only the consequence of the applied thermodynamic forces: in fact, the primordial cause in the formation of energy dissipative structures is the increase of the entropy production (equal to sum of the products of force times the current it produces) above a critical value that destabilizes the NESS’s belonging to the thermodynamic branch. The kinetic factors acting in such open systems are not the first-principles, i.e., the underlying thermodynamic principles, which explain the phenomena.^13^ Despite this misunderstanding, the reports on the reaction-diffusion topic belong to a plethora of works on important results in self-assembly and self-organization,^43−46^ but surely a change of hypothesis and models based on a more coherent irreversible thermodynamic scenario would lead to a significant progress in chemistry in all these topics that correspond to complexity, which are well accepted^47^ but in need of a genuine thermodynamic first-principles support. With respect to chemical complexity, we understand “self-assembly” as the spontaneous formation from simple building blocks of supramolecular structures, and self-organization as the dynamic coupling between supramolecular systems thanks to their specific chemical functionalities. These determine the exchange of chemical information (catalysis, coupled reactions, electrical charge changes due to electrolyte chemistry, etc.) between parts. It is worth noting that such an informational role of chemistry in complex systems^4,6,48^ is practically overlooked in application of physical methods to the study of biological complexity and Systems Biology (see section 5).

It is worth noting that the use of hydrodynamic flows is probably the only possible way to control the self-assembly and self-organization of nanoparticles and macromolecular species, as manual or robotic triage is unrealistic.^13,49^ In this respect, very important results have been achieved in microlithography^50^ and in applications of microfluidics.^51^ A less studied case is that of anisotropic particles of a molecular size already able to be oriented in a hydrodynamic flow because this gives rise to 3D anisotropic rate constants. In this respect, the breaking of chiral symmetry by effects either by the chiral shear forces of a chiral hydrodynamic flow exerting a chiral sign selection by a top-bottom size scale transfer of mechanical forces, or perhaps because of anisotropic/chiral mass transfer growth, has been reported.^40,52−56^

## 4 New Elements Required for a Teaching Scenario on Irreversible Thermodynamics for Chemistry: Chemical Reactions in Open Systems

Pedagogical concepts and desirable simple models, supported by physicochemical principles of nonlinear irreversible thermodynamics, are so necessary for the support of chemical experimentation that they should be commonly used by the upcoming 2050 horizon. An obstacle for this goal is the current chemical syllabus exclusively centered in the completeness of reversible thermodynamics, that is truly a complete theory but only when assuming unrealistic isolated systems: irreversibility is sometimes visualized as an uncomfortable fact destroying a perfect theory. The conceptual significance from the microreversible kinetic scenario to the statistical mechanical one describing the macroscopic irreversibility was a question already known to Gibbs and Boltzmann, and already formulated by P. and T. Ehrenfest in the year 1907.^57,58^ Obviously, there are textbook exceptions to this, with the seminal example being the book by Kondepudi and Prigogine.^28^ For example, most of the successful textbooks do not clearly address the importance of irreversibility in natural phenomena and in applied chemistry. Instead, (a) the discovery of irreversibility by the disagreement between the results from molecular mechanics of gas kinetics and its statistical mechanics representation is presented as the description of entropy but without stressing the difference between microscopic and macroscopic worlds, and (b) the consideration of activities instead of concentrations in the definition of chemical potentials is presented as the crucial characteristic of real systems, despite the fact that this does not change the microreversible scenario of the model.

### 4.1 General Evolution Criterion (GEC)

Chemical networks in far from equilibrium settings can not only be described by the reaction network but also by the boundary conditions and are prime examples of how complex systems operate and evolve following the dictates as set down by nonequilibrium thermodynamics.

**Figure 1:**
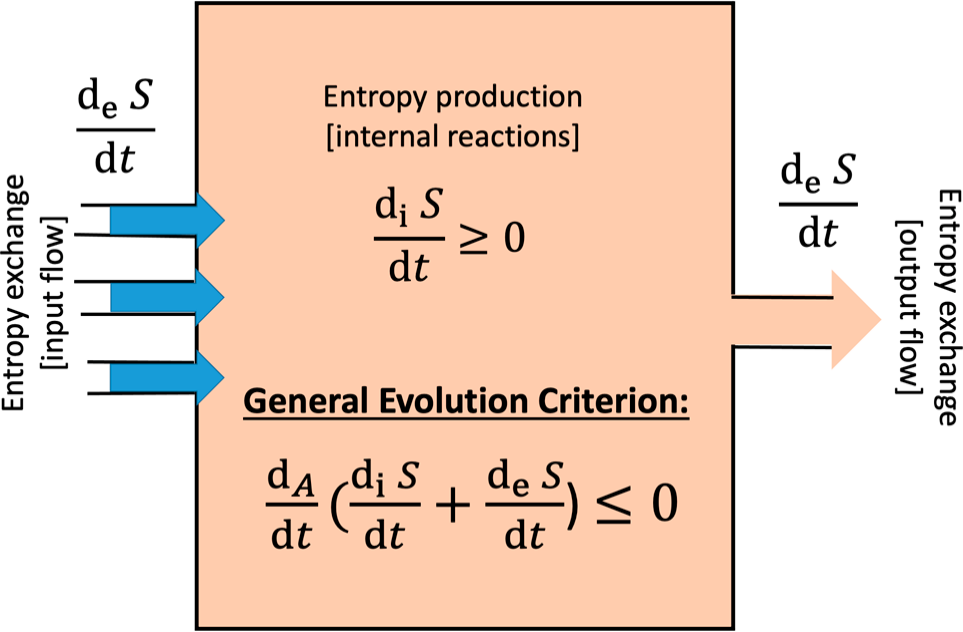
Schematic representation of a continuous flow stirred tank reactor (CSTR) as an example of how the entropy production and the entropy flows balance in a system exchanging matter with the environment. Species flow in at fixed concentrations (left), all species flow out with their instantaneous concentrations (right) as determined within the reactor. The internal microreversible reactions lead to a positive definite entropy production d_i_*S*/d*t* ≥ 0, while the input/output matter flows lead to entropy exchanges d_e_*S*/d*t* (these can be either positive or negative) with the surroundings. The general evolution criterion (GEC) is the statement that the change, of the sum of the entropy production plus the total entropy exchange, with respect to the temporal derivative of the forces (the chemical affinities, A) is negative semidefinite and is strictly zero at a NESS.

In particular, the entropy production or the rate of dissipation, the entropy exchanged between system and its environment, as well as the overall balance of entropy at a stationary state, are fundamental concepts in the thermodynamic characterization of far-from-equilibrium open systems because they consider the irreversibility of natural phenomena, as exemplified by chemical and biochemical reactions, and by the functioning of biological cells. Moreover, a general inequality for the evolution of the entropy production, valid for the entire range of macroscopic physics and chemical reactions and for fixed boundary conditions, was established originally by Glansdorff and Prigogine.^10,59^ We have extended this general evolution criterion (GEC), so as to include well-mixed open-flow reaction systems.^60^ This recent result yields an inequality constraining the time rate of change of the entropy balance: namely, the sum of the entropy production and the exchange entropy, or entropy flux, see Figure 1 and Scheme 1 and below for the extended GEC. Now, the entropy production σ is a product of thermodynamic forces *F*_α_ and currents *J*_α_, and is always positive definite:
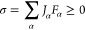
1

For reversible chemical reactions in open-flow and well-mixed chemical reacting systems, the forces are expressed through the affinities
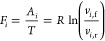
2and the currents are
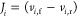
3where the forward (f) and reverse (r) absolute rates of the ith reaction are *v*_*i*,f_ and *v*_*i*,r_.

The GEC theorem of Glansdorff and Prigogine says that the derivative of σ with respect to the temporal changes in the forces *F*_α_ obeys the inequality
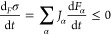
4with strict equality to zero holding at any stationary state.

The fundamental importance of the extended inequality (see Figure 1 and Scheme 1) is that it governs the joint evolution of the microreversible reactions taking place within the reactor volume together with the one-way, irreversible input/output matter fluxes that couple the system with its environment (the boundary conditions). The GEC governs the way that the chemical forces, or affinities, can change in time and, most importantly, how the change in the affinities governs the evolution of the system’s entropy production and exchange entropy. The range of validity and applicability of the GEC encompasses systems for which the assumption of local equilibrium holds (see discussion in section 4.2 below and also Chapter II.2 of ref (10) for further details). Notice that at thermodynamic equilibrium as well as in the linear regime of irreversible thermodynamics, and due to the linear and sign relationships between the forces *F* and their currents *J*, one has , so that from the identity and using eq 4, eq 5 simplifies to yield the celebrated theorem of minimum entropy production (TMEP)^10^
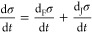
5


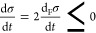
6

Notice that TMEP in chemical reactions can be described by expression 6, when *A*/*RT* ≪1, also from the Onsager mathematical symmetrical linearity between forces and currents, because the absolute reaction rate ***v***_***r***_ at the NESS (see section 16.5.1 of ref (28)) can be expressed as
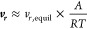
7

This theorem probably helped to overshadow the deeper significance of the GEC, which states that the dynamic evolution of the system does not follow a linear relationship between forces and currents, but does imply the thermodynamic forces must change in time in such a way as to tend to decrease the rate of entropy production toward a minimum value (see pp 163–167 of ref^61^). Probably this relationship between the rate of entropy production and the dynamic evolution of the system will be described pedagogically by the 2050 horizon as a universal friction working against entropy production that would explain how NESS’s can become unstable and how thermodynamic limits are established to an otherwise permanent and unbounded entropy growth. Growth of entropy cannot increase without limit, such as suggested in the student mantra that entropy always increases. The GEC implies the slowing down of the rate of dissipation or production of entropy. This, in the linear regime of irreversible thermodynamics, is expressed by the Theorem of Minimum Entropy Production (TMEP)^28^ and in the nonlinear regime by the GEC. That the GEC reduces to the TMEP for NESS close to equilibrium follows from an indistinguishable departure from linearity between the forces and currents (see Scheme 1).

**Scheme 2:**
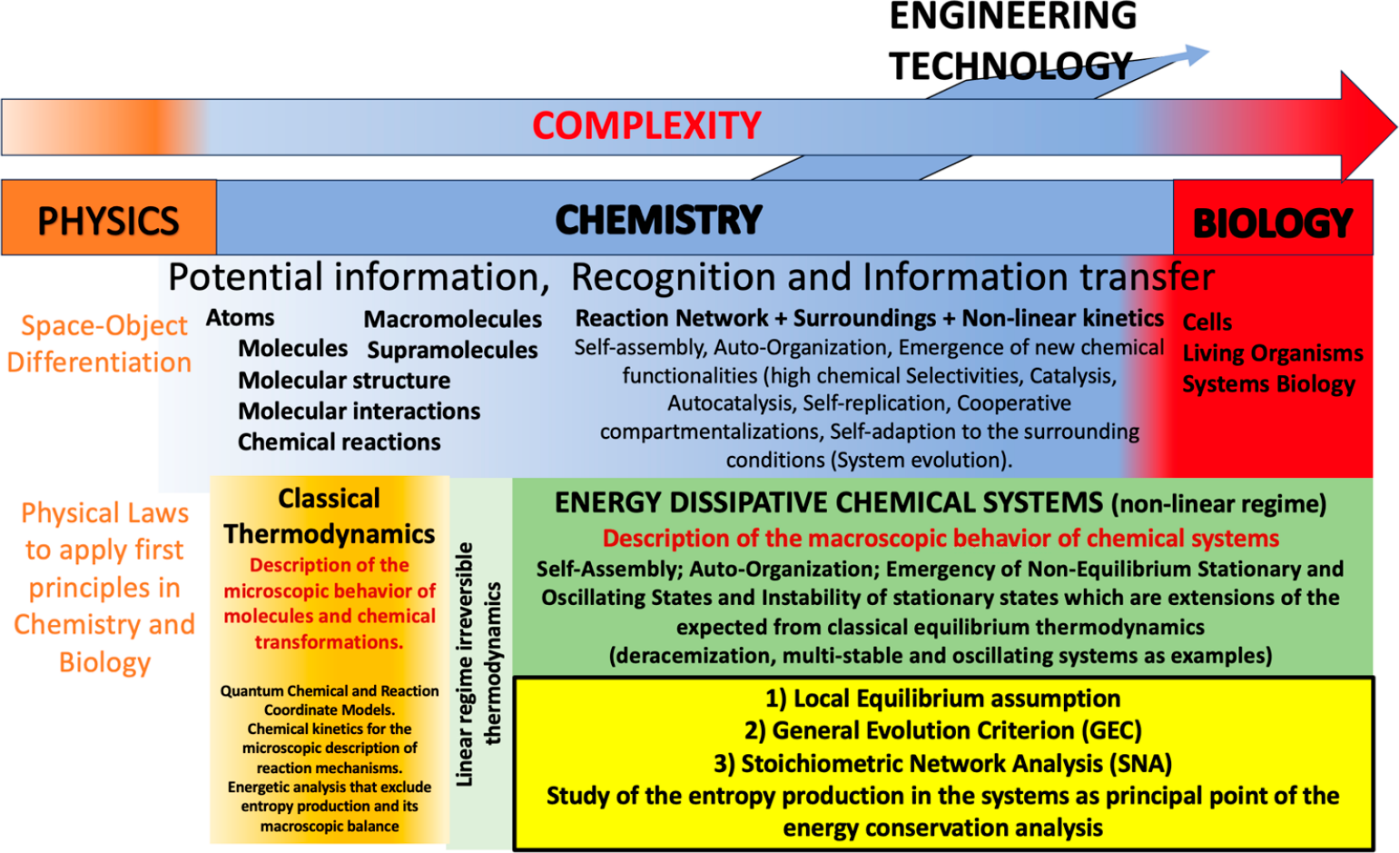
Chemistry as a Natural Science Spans a Tremendous Epistemological Gap between Physics and Biology To explain, in understanding results and design experiments, the conceptual and instrumental agreement with the scenario of Energy Dissipative Systems is necessary and whose thermodynamic rules are those of the nonlinear regime of irreversible thermodynamics. The latter is a mature and well-developed field of thermodynamics.

Therefore, we could imagine how thermodynamics could be presented from the more general case of irreversibility (4) down to the linear regime (6) and finally to the thermodynamic equilibrium, where the balance between internal entropy production and external entropy flow is simplified by the isolated system scenario (no flow). By way of an explicit example, we illustrate and validate the extended GEC for the bistable Schlögl model subject to open flow in section 4.4.2.

### 4.2 Local Equilibrium Assumption

The important hypothesis underlying nonequilibrium thermodynamics is the local equilibrium hypothesis.^10,28,62,63^ For many macroscopic systems, we can assign a temperature *T* and other thermodynamic variables to every elemental volume Δ*V*, and assume that the equilibrium thermodynamic relations are valid for the thermodynamic variables assigned to each such elemental volume. The local and instantaneous relations between thermodynamic variables in each individual Δ*V* belonging to an out-of-equilibrium system are the same as for a uniform system in equilibrium. This is the concept of local equilibrium.

A consequence of the local equilibrium assumption is that all the intensive and extensive variables defined in equilibrium such as entropy, energy, temperature, chemical potential, and so forth, are defined out of equilibrium, but they are now allowed to vary with time and space, and so become functions of the latter. A further consequence is that the local state variables are related by the same state equations as in equilibrium. This means, in particular, that the Gibbs’ relation between entropy and the state variables remains locally valid for each value of the time *t* and the position vector **x**. Assume the local entropy *s* is the same function of internal energy *u*, specific volume *v*, chemical potential μ_*k*_, and mass fractions *c*_*k*_, as in equilibrium. Then we can write the local Gibbs relation as follows:
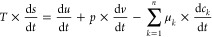
8

The local equilibrium hypothesis states that at a given instant of time, equilibrium is achieved in each individual and elemental volume cell Δ*V*. Of course, the state of local equilibrium is different from one elemental cell to another, and so mass and energy exchange is allowed between adjacent cells. In each individual cell, the equilibrium state need not be stationary but can change in time.

The physical conditions that make local equilibrium a valid assumption can be understood in terms of the separation between the time scales of collisions and of the macroscopic processes. The former time scale, τ_m_, denotes the equilibration time inside one elemental cell Δ*V* of the primary event, necessary but not sufficient, that precedes the chemical transformation: for example, two successive collisions between particles in a second-order molecular transformation or the vibration/oscillation proceeding the breaking of a chemical bond. This, in the Arrhenius equation, is expressed by the pre-exponential factor (*A*_Arr_), that typically is a very large number. The second characteristic time scale τ_M_ is a macroscopic one whose order of magnitude is related to the duration of the macroscopic process under study. This, in the Arrhenius equation, is expressed by the rate constant (*k*), that, because the activation energy for common chemical process and common temperature ranges is exp{−*E*_a_/*RT*} ≪ 1, implies the rate constant is an order of magnitude smaller than the exponential prefactor *A*_Arr_. Defining the ratio between both reference times by , in almost all reactions only a very small fraction of the molecular collisions are able to produce the chemical transformation. Most of the reaction rates encountered in laboratory chemistry indicate that reactive collision rates are several orders of magnitude smaller than the overall collision rates. Between reactive collisions, the system quickly relaxes to equilibrium, redistributing the change in energy due to the chemical reaction. Thus, any perturbation of the Maxwell velocity distribution due to a chemical reaction quickly relaxes back to the Maxwellian one with a slightly different local temperature. Hence, on the time scale of chemical reactions, the temperature is locally well-defined.

In summary, for *De* ≪ 1, the common chemical scenario, the local equilibrium hypothesis is fully justified because the relevant macro-variables evolve on a large time scale τ_M_ and practically do not change over the shorter time scale τ_m_. Notice that these time scales can be qualitatively estimated by chemical reasoning for any chemical process. Nevertheless, the hypothesis is not appropriate for describing situations characterized by *De* ≥ 1. This will occur in the case of reactions with extremely low activation energies. This is because τ_M_ and τ_m_ will then have similar values, as for example in the case of the irreversible transformations in gas pyrolysis, ultrasound propagation, shock waves, nuclear collisions, (τ_M_ is very short). Furthermore, *De* ≈ 1 can also occur in systems with long primary relaxation times, i.e., having small prefactor *A*_Arr_ values, as for example in polymer stereochemical transformations or gel dilation, for which τ_m_ is large and of the same order of magnitude as τ_M_. Convective and transport effects described by the Navier–Stokes equations are also within the domain of validity of the local description. On the contrary, shock waves and plastic deformations of solids lie outside the scope of this local equilibrium approach.

### 4.3 Entropy Production and Entropy Exchange in Chemical Reactions

We advocate that the approach to thermodynamics should be taught on (i) the basis of the entropy production, that is, the dissipation of energy due to irreversible processes taking place within the system and (ii) the flow or flux of entropy entering and leaving the system. The temporal changes in a system’s total net entropy *S* can therefore be expressed as a balance equation involving the sum of the entropy production and the entropy flux:^28^
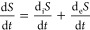
9where  ≥ 0 is the non-negative rate of entropy production due to the irreversible processes within the system and denotes the entropy flux due to the exchange of matter and energy between the system and the external environment (see Figure 1). The latter term can either be positive or negative and is strictly zero for the isolated systems of classical reversible thermodynamics. For microreversible chemical reactions, the rate of entropy production per unit volume  in well-mixed homogeneous systems can be expressed as a product of a force times a flow (see also eq 1):
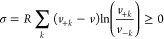
10in terms of the forward *v*_+*k*_ and reverse *v*_–*k*_ absolute reaction rates of the *k*th microreversible reaction and *R* is the universal gas constant.^28^

The entropy transported by the exchange flux with surroundings, called entropy exchange per unit volume , is given by^30^
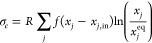
11

This depends on the concentrations of the species flowing into (*x*_*j*,in_) and flowing out from (*x*_*j*_) the reactor, with the volumetric flow rate *f* = *q*/*V*, where *q* is the volume of fluid per unit time entering and exiting the reactor. The equilibrium concentrations for the *k*th species *x*_*j*_^eq^ are determined from detailed balance and mass conservation: the stationary state corresponding to the reactor being isolated or “cut off” from the open flow *f* = *q* = 0. The chemical potential of the species at thermodynamic equilibrium is used to define the relative chemical potential μ_*k*_^rel^, which shifts the reference point of the standard chemical potential from the Gibbs energy of formation of compound *k* (μ_*k*_^0^) to the equilibrium state of the system as follows:^64,65^
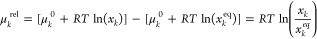
12

Notice that the expression 10 for the entropy production for a reversible reaction corresponds to the addition of the “partial entropy productions” corresponding to the two different single reactions, the forward and the backward reaction, where the chemical potentials are expressed by the corresponding relative chemical potentials such as in eq 12. Absolute rates are functions of the probability of the reaction in nonelastic collisions and of the chemical potentials of two independent reactions, forward and backward, and this is required for understanding the coupling inside complex reaction networks between individual transformations (see section 4.4.2).

The relative chemical potential is defined here for the case of ideal solutions where the activity is equal to the concentration. Note that the use of activities instead of concentrations does not change the points discussed here concerning the irreversible thermodynamic scenario. We emphasize that σ_*e*_ is an important aspect of open-flow reactors, essential for achieving entropy balance in nonequilibrium stationary states (NESS).^8,30,66^ The sum (σ + σ_*e*_) gives the entropy balance eq 9 per unit volume, , and the inequality presented below in Scheme 1 (see section 4.1) shows how the temporal behavior of entropy production and exchange is conditioned by the changes in the chemical affinities of the reactions and the pseudoreactions in the nonlinear regime of nonequilibrium thermodynamics.

Some comments concerning the validity of the GEC are warranted and to answer the first question (*i*) in section 3. The proof of the GEC appeals to the local equilibrium hypothesis, or assumption; see the above discussion and also section II.2 in ref (10). This assumption is valid for complex systems of chemical reactions with highly nonlinear kinetics with reaction rate constants on the order of chemical common processes. This implies a separation or decoupling of time scales between microscopic and macroscopic processes. Clearly then, in rare exceptional situations, where the local equilibrium hypothesis does not hold, we would then not expect the GEC to be valid but rather a generalization of it. Gradients would need to be included, and this is the subject of extended thermodynamics^29^ which, in view of the 2050 horizon, will remain as an active theoretical field of research.

The GEC gives no information concerning the putative stability of the nonequilibrium stationary states (NESS) in the nonlinear regime of nonequilibrium thermodynamics. This is because the GEC cannot be related in general to a kinetic potential. This is because the expression for the GEC involves a nonexact differential. On the other hand, the GEC does govern the way the generalized thermodynamic forces (the chemical affinities) evolve, and this force-evolution relationship is the scope of the GEC. In the original formal demonstrations of the GEC, equilibrium stability conditions are carried over to nonequilibrium thermodynamics by appealing to the local equilibrium hypothesis (see above). On the other hand, the stability question of the NESS is a major goal of studying the temporal dependence of the so-called excess entropy production, and its relation to Lyapunov functionals, a rather different problem, and these will probably be important topics in the physical chemical research publications during the next few decades.

### 4.4 Renewed Syllabus in Teaching Chemical Kinetics

The study of complex chemical systems requires, in addition to obtaining the mathematical solutions of complex sets of ordinary differential equations (ODE), identifying the states which represent the attractors of the system (stable NESS’s or types of oscillatory behavior instead of chaotic behavior), as well as finding the coupling between internal and exchange flows and how these determine the emergence of bifurcations when the thermodynamic branch becomes unstable. Notice that this methodological approach to describe a chemical reaction network through molecular potential energy surfaces by quantum chemical or molecular mechanics methods, despite continuous improvement in their accuracy and extension to complex sets of compounds,^67^ describes only microscopic states, which can be fairly extrapolated to macroscopic NESS’s represented by the thermodynamic branch (section 3). But these methods do not provide any reliable information about those NESS’s emerging in bifurcation scenarios. Furthermore, common methods based on the application of the chemical kinetics models are usually subjected to approximations and simplifications in order to find an analytical integration, whereas the use of numerical integration methods of the ODE sets are limited to the study of very complex reaction networks, mostly in chemical engineering applications.

#### 4.4.1 Applied Mathematics Support to the Kinetic and Dynamics of Complex Systems

Classical chemical kinetics searches for exact solutions of the ODEs to simulate the time evolution of chemical transformations and the composition of the final stationary states. As this is only possible for the simplest transformations, classical kinetics uses simplifications and approximations to express the boundary conditions, which fit to the framework of classical thermodynamics anchored in the isolated system description but that are crucial parameters in the behavior of open systems. Nowadays such approximations and simplifications are no longer necessary because of the universal and straightforward access to computers and mathematical-packages. Furthermore, there exist specific computing application packages for chemical kinetics, such as for example COPASI.^68^ However, these dedicated packages are limited by the fixed machine precision which presents a great limitation when solving, by numerical integration, ODEs containing rate constants that can differ among themselves by many orders of magnitude.^69^ This returns indirectly to some of the classical simplifications used in classic chemical kinetics. A consequence of this is that most chemical kinetics packages cannot describe networks involving the simultaneous presence of both extremely exergonic and endergonic reactions nor very fast (e.g., Brønsted acid/base proton transfer) and slow rate reactions (e.g., C–C bond forming/breaking reactions). Mathematical packages such as *Mathematica*,^70^ which allow one to perform the numerical integration far beyond the limitations of machine numerical precision, may avoid this common problem. In summary, a deeper understanding of applied mathematics for numerical integration computing methods to increase the expertise of future chemists would be highly desirable in future syllabuses.

#### 4.4.2 Matrix Description of the Reaction Networks: Stoichiometric Network Analysis (SNA)

The matrix representation of the global differential equations describing the reaction networks reduces the complexity of the system to more manageable matrix operations leading to solutions for ranges of the system’s parameters. Notice that the study of chemical bifurcations requires the inspection of diverse sets of the systems parameters, and that this is possible through such a general matrix description of the system. Such a matrix methodology to describe chemical systems was developed by Clarke in 1980.^71−75^ The method is based on the formalism of the representation of a reaction network by the matrix resulting from the multiplication of a stochiometric matrix (**S**, formation or elimination of each species in each transformation) by the rate vector (, kinetic order dependence of each species in each reaction). The method allows the recognition of the emergence of NESS’s beyond the thermodynamic branch.^74,76^ Further, it describes the irreducible currents which make up the reaction network, and this helps to understand the coupling between the environment and the internal reaction network.^30,66^ Unfortunately, for chemists, it has the disadvantage of not being intuitive when compared with commonly used chemical methods. However, this is probably more a consequence of the strong contrast with the present methods used for teaching chemical kinetics. This means that something could be changed by improving pedagogical approaches to the mathematical description of chemical transformations.

We review the basic elements of SNA.^73^ We begin with the chemical reactions for *r*-reactions involving *n* reacting species obeying mass-action kinetics:
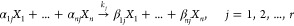
13where the *X*_*i*_, 1 ≤ *i* ≤ *n* are the chemical species and *k*_*j*_ is the reaction rate constant for the *j*th reaction. We consider all the chemical transformations as one-way irreversible transformations. A reversible reaction is therefore decomposed into two independent mass action controlled forward and reverse reactions. The matter flow terms defining the matter exchange of the open system with the environment are treated as irreversible one-way “pseudoreactions” and are easily incorporated into this list (eq 13). Then from eq 13, we read off the entries of the *n x r* stoichiometric matrix **S**:
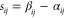
14

For mass-action kinetics, the reaction rate ν_*j*_ of the *j*th reaction is a monomial,
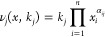
15and where the *x*_*i*_ = [*X*_*i*_] are the concentrations.

The differential kinetic rate equations corresponding to the set of reactions (including the input/output flow terms) in eq 13 can be written in condensed matrix form,^77^
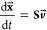
16

**Figure 2:**
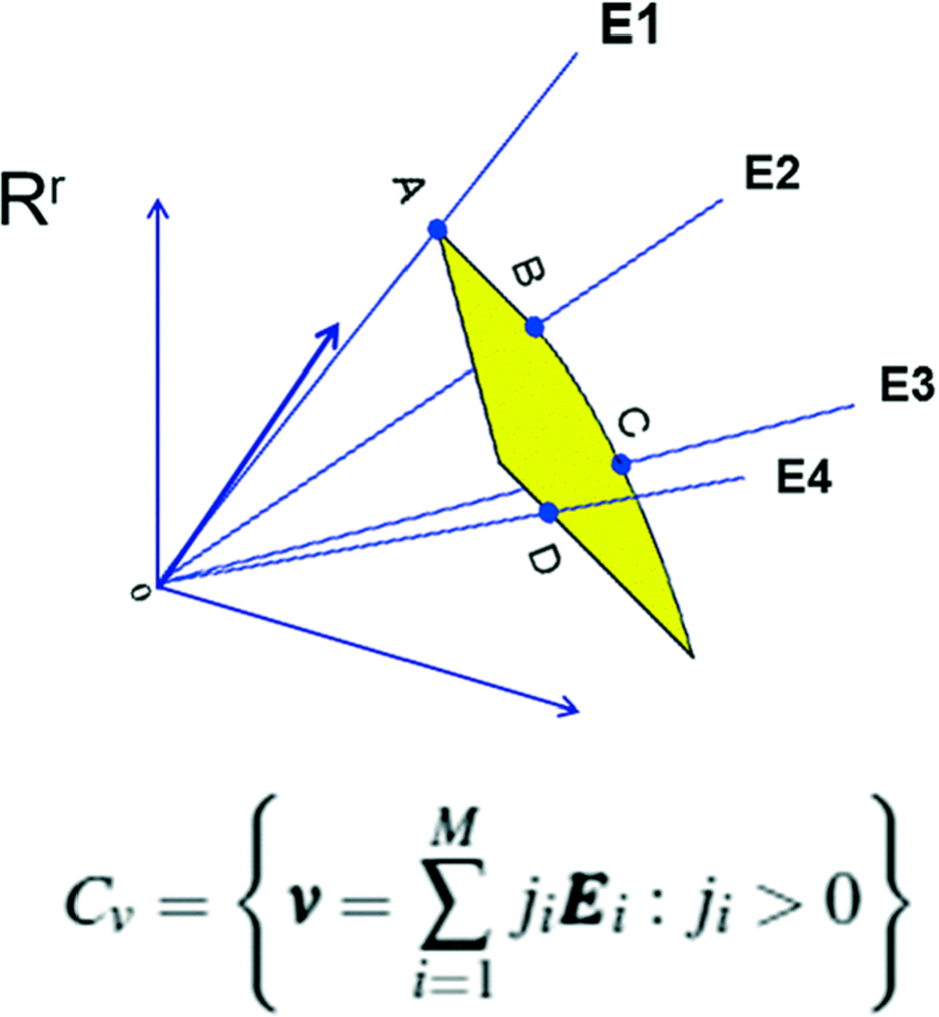
Convex cone ***C***_***v***_ lies in the positive orthant of an r-dimensional Euclidean space *R*^*r*^. The dimension *r* is the number of individual one-way reactions, where the forward and backward reactions are considered to be independent.

Just like the stoichiometry, the chemical pathway structure should be an invariant property of the reaction network.^73^ This pathway structure follows from the steady state condition **0** = **S**, which also defines the right null space of the stoichiometric matrix **S**, and corresponds to the set of all stationary solutions of eq 16. But since the reaction rates of eq 15 are positive-definite ν_*j*_(*x*, *k*_*j*_) ≥ 0, for all *j*, they must therefore belong to the intersection of the right null space of **S** with the positive orthant of an r-dimensional Euclidean space: **R**_+_^r^ (a multidimensional space determined by the number of one-way reactions). This intersection defines a convex polyhedral cone *C*_ν_ which is spanned by a set of *M* generating vectors, or elementary flux modes (EFM), which give the irreducible vectorial representation of the reaction network, **E**_i_ for *i* = 1,2,···,*M* (see Figure 2). The convex cone is the set of all linear combinations (with positive coefficients *j*_*i*_ > 0) of these **E**_i_:
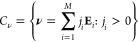
17

The EFM vectors **E**_i_ have r-components equal to the number of unidirectional reactions in eq 13 and they point along the *M* edges of the convex cone *C*_ν_ (see a schematic simplification in Figure 2). These EFM vectors **E**_i_ can be easily obtained using the freely available COPASI program.^68^ The **E**_i_ corresponds to subsets, or combinations, of several of the unidirectional reactions in eq 13 in an *r*-dimensional Euclidean space. Some of these EFM vectors may involve the coupling of the pseudoreaction fluxes with the chemical transformations and need not necessarily include simultaneously both the forward and reverse steps of the same chemical reaction. The angles between the EFM vectors gives the degree of coupling between the corresponding EFM chemical currents; orthogonality between two cone edge vectors implies that the sets of reactions represented by them are uncoupled or independent. A general stationary reaction rate vector , or steady-state flux, is represented as a point in this cone and is expressed as a positive linear combination of these cone edge vectors, eq 17. The positive expansion coefficients *j*_*i*_ > 0 are called the convex parameters, and they give the magnitudes of the matter fluxes along the specific chemical pathway represented by **E**_i_. The set of all the possible steady state fluxes are represented by this convex cone ***C*_ν_**, see Figure 2 for a schematic representation.

A remarkable advantage offered by SNA is that it can also be used to analyze the entropy production in open chemical systems. This is because the so-called partial dissipation along each individual extreme flux mode (EFM) can be defined and calculated. This feature allows one to determine the contribution from each pathway (or EFM) and so identify which reaction pathways are the relevant ones leading to the symmetry breaking and for the onset of dissipative structures in the case of spatially inhomogeneous systems. In other words, SNA gives a path-oriented approach for understanding entropy production and the GEC.^30,60,66,78^ Furthermore, it gives a quantitative and rigorous explanation as to why the flow of energy can lead to a decrease of entropy production, as in the case of symmetry breaking instabilities, or else in other situations it may increase the entropy production, as in the case of the Bénard instability. In the former case, in symmetry breaking, the number of EFMs decreases, hence there is less dissipation compared to the symmetric phase. In the latter example, this is because the emergence of new spatial structures implies new mechanisms of dissipation which are not present before the instability. So, entropy production can either increase or decrease, and which outcome pertains depends on the nature of the instability. In far from equilibrium nonlinear systems, there is no rigorously established rule, theorem nor “principle” of either maximum nor minimum entropy production, but only the increase or decrease of the number of pathways, and mechanisms which itself depends on the instability involved and on the boundary conditions of the system under study.

SNA is being used in the description of complex biochemistry cycles.^79,80^ A technical problem is that highly complex systems yield a large number of EFM vectors, so that pattern recognition computing methods are necessary for identifying the crucial couplings between reactions that determine the emergence of the attractors. This means that work in the topic would require a wider spread of expertise in computing methods than is the case in chemical research at the present time.

We have reported on the application of SNA to the study of the NESS appearing in bifurcation scenarios leading to the instability of the NESS on the thermodynamic branch. See examples of the selectivity between enantiomers leading to the deracemization of racemates (spontaneous mirror symmetry breaking, SMSB) in refs (30, 60, 66, and 81). In the following, an example is given of the powerful tool that SNA and the knowledge of the EFMs represent in the so-called Schlögl model: it allows one to understand how, for some set of system parameters, the thermodynamic branch NESS’s can become unstable and two other stable NESS’s act as attractors for the system’s evolution.^41^

##### 4.4.2.1 Schlögl Bistable System As Example of the Use of SNA and the Use of EFM for the Study of Dissipative Systems

**Figure 3:**
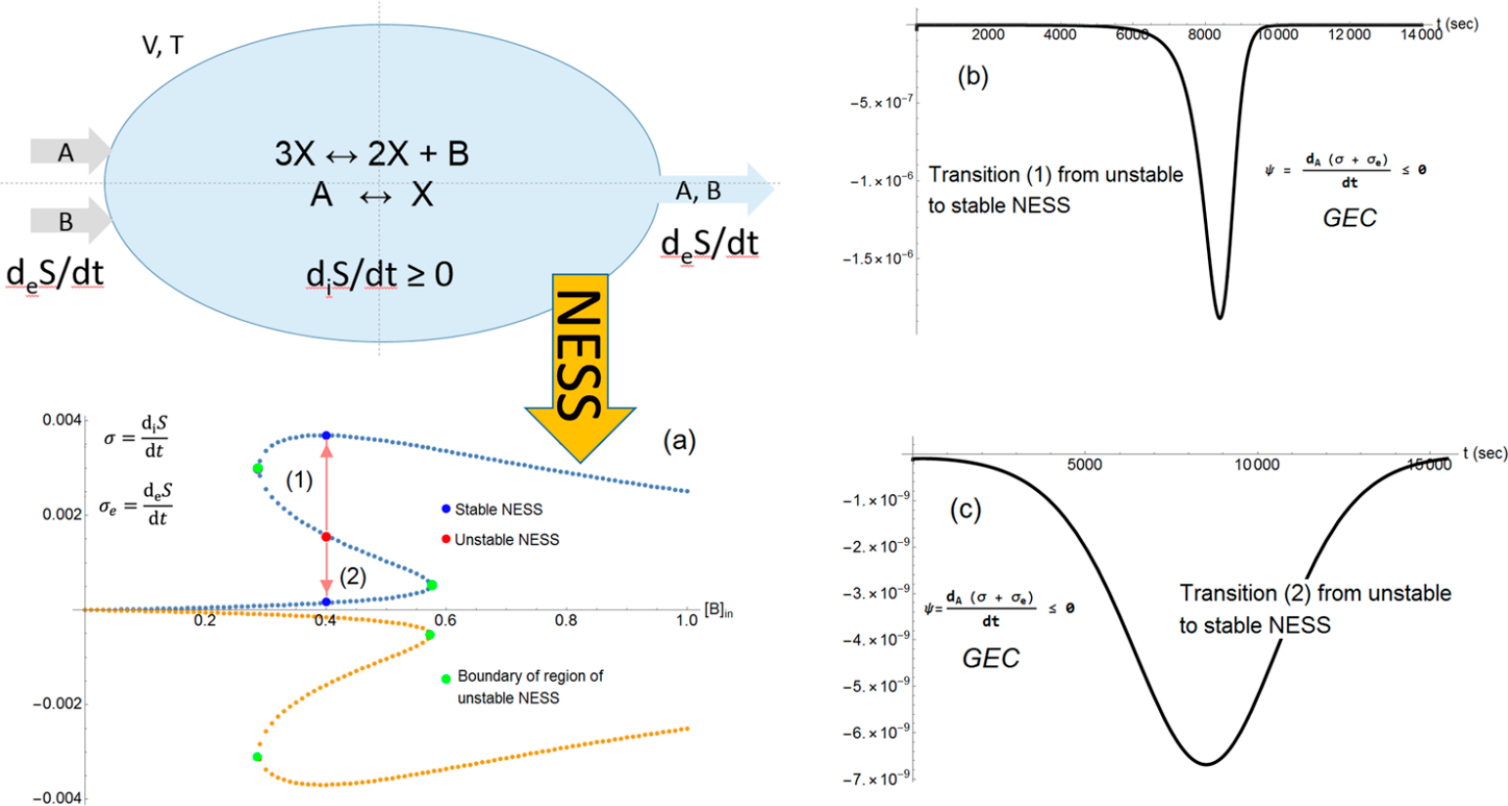
Validation of the extended general evolution criterion (GEC) for dynamic transitions in an out-of-equilibrium bistable chemical model. Upper left side: reversible reactions defining the Schlögl model in an open-flow well-stirred and isothermal (*T*) reaction tank of volume *V*. Species A and B show entry flows in at fixed concentrations [A]_en_ and [B]_en_, respectively. In the model illustrated here, both A and B, but not X, flow out with their instantaneous internal concentrations as determined by solutions of the differential kinetic rate equations. (a) Upper blue curve shows the entropy production per unit volume σ, and the lower ochre curve is the exchange entropy per unit volume σ_e_. Both are evaluated at the NESS’s as functions of the fixed input concentration [B]_en_. Entropy production and exchange are balanced on all the NESS’s: σ + σ_e_ = 0 (units J^–1^ K^–1^ s^–1^ L^–1^). The region of unstable NESS is bounded by the pair of green dots. Transition (1): from the unstable NESS located at [B]_en_ = 0.4 (red dot) to the stable NESS (blue dot) of greater entropy production on the upper stable segment, alternatively, transition (2) represents the evolution to the stable NESS (blue dot) of the lesser entropy production located on the lower segment of stable NESS. (b) Evaluation of the extended GEC for transition (1). (c) Evaluation of the extended GEC for transition (2). Both transitions (1) and (2) from the unstable to the stable NESS obey the GEC, and which alternative path is chosen is determined solely by the sign of an initial compositional fluctuation, such as, e.g., ± δ[A]. Recalculated from data of ref (78).

The chemical Schlögl model is a simple autocatalytic reaction scheme possessing a first-order nonequilibrium phase transition and hysteresis.^82,83^ See Figure 3 for the transformations involved and reactor configuration. SNA shows that the elementary flux modes (EFM), i.e., the couplings between specific one-way reaction flows and the matter exchanges with the surroundings, are as follows:
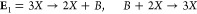

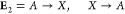

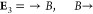

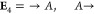

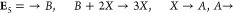

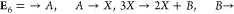


**E**_1_ and **E**_2_ correspond to the two reversible reactions that define this model, whereas **E**_3_ and **E**_4_ represent the unreactive flow-through from the input to the output of B and A, respectively. The EFM **E**_5_ represents the sequence of the two reverse reactions driven by the input of B and the output of A. The EFM **E**_6_ represents the sequence of the two forward reactions driven by the input of A and the output of B. The overall pathways that **E**_5_ and **E**_6_ represent are traversed in opposite flow directions: either from B (in) to A (out), or else from A (in) to B (out), and both these open pathways are productive. The opening of the Schlögl model to such matter flows gives rise precisely to these two latter EFMs and which are completely absent from the clamped version. Multistability and bifurcation analyses can be carried out in terms of the six positive convex parameters, see ref (78) for details.

The model was originally defined and analyzed under the restrictive assumption of a single time dependent species X involving two clamped species: A and B. Here, by contrast, we consider the model for which all three species A, B, and X are allowed to vary with time in an open-flow reactor; see upper left-hand side of Figure 3. We perform the calculation of  and validate the GEC for the dynamic transitions (illustrated for input concentration [B]_en_ = 0.4) starting off on the corresponding unstable NESS and ending up on one of two stable NESS. Figure 2b shows the dynamics and the confirmation of the GEC for the transition to the stable NESS located on the upper portion of the entropy production curve (see panel a). Figure 2c shows the dynamics and the GEC for the transition to the other stable NESS located on the lower portion of the entropy production curve. The behavior of the transitions from the interval of the unstable NESS to either one of the two alternative stable NESS’s is qualitatively similar to the results displayed here. The important qualitative feature is that the expression for the GEC starts off zero on any initial NESS. A compositional fluctuation then moves the system away from an unstable NESS and the expression is strictly negative definite as the system evolves irreversibly to the final stable NESS and goes to zero asymptotically when the system approaches that stable NESS. A positive fluctuation in concentration added to either the A or to the X species or else a negative fluctuation in concentration added to B leads to results qualitatively like those shown in Figure 3b. On the other hand, a negative compositional fluctuation added to either A or to X, or else a positive fluctuation added to B, leads to results qualitatively like those shown in Figure 3c. The GEC has been confirmed for all these individual cases (not shown). That is, starting off the system on any one of the unstable NESS located on the hysteresis section of the curve, see panel (a). The GEC is obeyed for all the allowed transitions between the unstable and stable NESS.

The GEC can also be used to distinguish between the alternative dynamic transitions from the unstable NESS to one or the other stable NESSs. This is so because the integrated dissipation due to the changes in the chemical affinities suffered along each individual transition is distinct: this integral is path-dependent. An estimation of this quantity is provided by , where *t*_*i*_ and *t*_*f*_ denote initial and final times of the transition, respectively, and these time scales depend on the pair of initial and final NESS’s, as does the integrand itself. Panels (b) and (c) in Figure 2 illustrate graphically how the initial and final transition times as well as the shape and the minimum value of the GEC curves depend on the transition between the specific pair of unstable and stable NESS.

## 5 Concluding Remarks

In contrast with the old scenario from the beginning of the XX century, chemistry no longer leads the way in the knowledge and technological advance of human progress. This is probably due to the exhaustion of the classical topics of solution and synthetic chemistry. Scheme 2 suggests that the role of chemistry as a natural science is to establish a bridge between Physics and Biology, the span of which involves broad and diverse unexplored fields. There are efforts in this direction, for example in the development of the so-called topic of systems chemistry.^84,85^ To close the tremendous gap between chemistry, as a natural physical science, and biology, as a science of complex chemical-based systems displaying functionalities (living-state systems) as well the different ramifications that should arise toward engineering and technological fields would change radically the topics and objectives of the future reports in physical chemistry.

In our opinion, this is due to the lack of chemical methodology and the chemist’s expertise for understanding, interpretation, and experimental design of work in open systems. Open systems are in fact the genuine and common ones, with their coupling relationships between internal reaction networks and boundary conditions. Driven by scientific need, we foresee that the pedagogical approach for teaching and learning physical chemistry will change dramatically within the next 50 years to explain the emergence, by self-assembly and auto-organization in open chemical systems, of chemical complexity and chemical functionalities. This important change in physical chemistry curricula will consist of the division of the present syllabuses into three well-differentiated blocks. First, (a) new chemical kinetics based on applied mathematics for the numerical simulation of chemical networks and their matter/energy exchange with surroundings with emphasis on the analysis of nonlinear dynamics. Second, (b) the actual body of classical reversible thermodynamics presented as laws and the historical description of how state functions were discovered, and the laws that relate one to another. And third, (c) the interaction between chemical species with the boundary conditions in irreversible processes, but under the thermodynamic constraint of their chemical and electrochemical potential, and how complexity and self-organization arise thanks to the balance of entropy production with the entropy flows and currents. The first block will make use of and benefit from numerical simulation of nonlinear reaction rate equations employing available high-precision mathematical programs and packages.

## Abbreviations

EFM: elementary flow modes
GEC: general evolution criterion
NESS: nonequilibrium stationary state
SNA: stoichiometric network analysis
TMEP: theorem of minimum entropy production
